# Water Dynamics at the Solid–Liquid Interface to Unveil the Textural Features of Synthetic Nanosponges

**DOI:** 10.1021/acs.jpcb.9b11935

**Published:** 2020-02-18

**Authors:** Paolo Lo Meo, Federico Mundo, Samuele Terranova, Pellegrino Conte, Delia Chillura Martino

**Affiliations:** † Department of Biological, Chemical and Pharmaceutical Sciences and Technologies (STEBICEF), University of Palermo, V.le delle Scienze ed. 17, 90128 Palermo, Italy; ‡ Department of Agricultural, Food and Forest Sciences (SAAF), University of Palermo, V.le delle Scienze ed. 4, 90128 Palermo, Italy

## Abstract

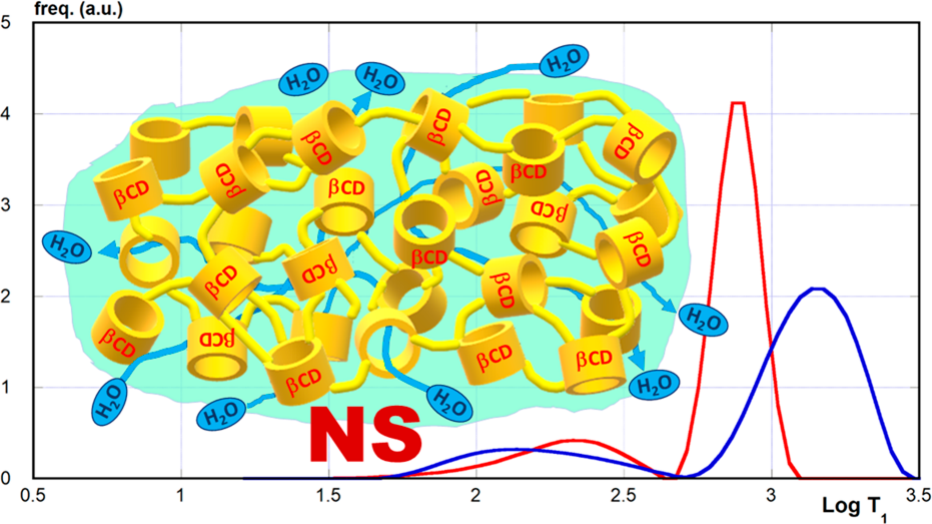

A fast-field-cycling NMR investigation was carried out on a set of polyurethane cyclodextrin nanosponges, in order to gain information on their textural properties, which have been proven to be quite difficult to assess by means of ordinary porosimetric techniques. Experiments were performed on both dry and wet samples, in order to evaluate the behavior of the “nonexchangeable” C-bound ^1^H nuclei, as well as the one of the mobile protons belonging to the skeletal hydroxyl groups and the water molecules. The results acquired for the wet samples accounted for the molecular mobility of water molecules within the channels of the nanosponge network, leading back to the possible pore size distribution. Owing to the intrinsic difficulties involved in a quantitative assessment of the textural properties, in the present study we alternatively propose an extension to nanosponges of the concept of “connectivity”, which has been already employed to discuss the properties of soils.

## Introduction

Nanosponges (NSs)^1−3^ are an emerging class of smart materials. Their tunable absorption and release abilities toward both organic^4^ and inorganic^5^ species make them ideal candidates as platforms for drug carrier/delivery systems,^6,7^ environmental remediation devices,^8,9^ and supports for metal nanoparticle catalysts.^10−13^ These materials are obtained by reacting supramolecular host units (e.g., cyclodextrins,^14^ calixarenes,^15^ pillararenes^16^) with suitable reticulating agents that afford the linker units. The properties of NSs can be widely tuned by either premodification of the host monomers or postsynthesis chemical modification of the obtained product.^17^ Moreover, reticulating agents bearing ionizable (polyamines,^18^ triazoles,^19^ pyromellytic anhydride^20,21^) or other stimuli-sensitive^22^ groups can be used; hence, functional tailored materials can be obtained.

A reliable evaluation of the textural features, such as average pore size (*D*), specific surface area (*S*), and specific pore volume (*V*), constitutes one of the main issues in the characterization of NSs, in order to rationalize their properties. Because of their peculiar hyper-reticulated highly disordered structure, NSs are supposed to present a thick network of nanosized channels between the host monomers. However, it has been reported on several occasions that the ordinary methodologies based on the N_2_ gas adsorption isotherms, analyzed by the well-known BET^23^ and BJH^24^ approaches, result in abnormally low values (sometimes even below the instrumental sensitivity limits), unless particularly long and rigid structural units are used as the linkers. For instance, *S* values ranging up to 263 m^2^ g^–1^ were claimed for a series of materials obtained from β-cyclodextrin (βCD) and tetrafluoro-terephtalonitrile.^25^ Comparable results were found by using decafluorobiphenyl,^26^ whereas an outstanding area of 1225 m^2^ g^–1^ and a pore volume of 1.71 cm^3^ g^–1^ were found for a material obtained by reacting a *per*-benzyloxy-cyclodextrin with formaldehyde dimethylacetal.^27^ Nevertheless, beyond these few exceptions, *S* values by far below 10 m^2^ g^–1^ are usually found for materials prepared from cyclodextrins and typical reticulating agents such as diisocyanates,^28,29^ epichlorohydrin,^30,31^ or poly(carboxylic acid)s.^32,33^ Similarly, *S* values not larger than 8 m^2^ g^–1^ were found for a series of cyclodextrin–calixarene copolymers, together with *V* values smaller than 0.03 cm^3^ g^–1^ and *S* values smaller than 4 nm.^34^ Wilson et al. recently examined a set of polyurethane βCD polymers obtained with diverse reticulating diisocyanates.^35^ They studied the relevant textural properties by both the ordinary BET method and a different approach based on the absorption of a suitable probe dye, namely, *p*-nitrophenol, having a known molecular area (0.525 nm^2^) and volume (0.0908 nm^3^). The latter methodology relies on the evaluation of the maximum absorption capacity of the material, obtained by analyzing the absorption isotherms by either the Langmuir or the Sips model. It led to the estimation of *S* values on the order of several hundreds of m^2^ g^–1^, which undoubtedly appears as a much more sensible result, in comparison to N_2_ adsorption. However, even this method is not devoid of criticism (see later), because the results obtained critically depend on the choice of the probe molecule and can be unpredictably affected by rearrangements of the microscopic structure due to swelling.^35^ Therefore, it seems that the concept itself of “surface” has conceivably a somehow elusive or deceptive meaning in the case of NS materials, so that new paradigms should be possibly explored.

Since the seminal work by Brownstein and Tarr,^36−38^ fast-field-cycling (FFC) NMR relaxometry has been used as a valuable tool to assess water mobility at the liquid–solid interface (and also to study the microscopic dynamics of polymers^39−44^ and proteins^45−48^). Data interpretation can be related to the textural features of solid systems such as clays and microporous materials in general.^49−59^ Very briefly (the bases of FFC-NMR relaxometry are summarized in the Supporting Information), this technique relies on the simple though counterintuitive idea that the tighter a water molecule is bound to the surface of a porous wet system (that is the more restricted its motion is), the faster the longitudinal relaxation rate (*R*_1_) is that the water ^1^H nuclei will experience (the same is true, indeed, also for transverse relaxation, which we do not consider here). Thus, as long as water is trapped into micropores, mesopores, or macropores, the observed *R*_1_ values decrease in the same order.^60^ Therefore, FFC-NMR relaxometry can also be used as a valid alternative to the traditional porosimetry investigations to obtain pore size distributions.^60^ Very recently, FFC-NMR relaxometry has been also applied in soil science^61^ to quantitatively measure the hydrological connectivity inside the soil (HCS).^62,63^ In general, connectivity refers to the processes involving a transfer of matter, energy, and/or organisms within or between elements of an ecological system. This implies the presence of a transport vector, such as water, and accounts for how mobility within the spatial patterns inside the soil (referred to as the “*structural*” connectivity) affects the occurrence of physicochemical processes (subsurface flow, sediment transport, etc., which is referred to as the “*functional*” connectivity).^62,63^

**Figure 1:**
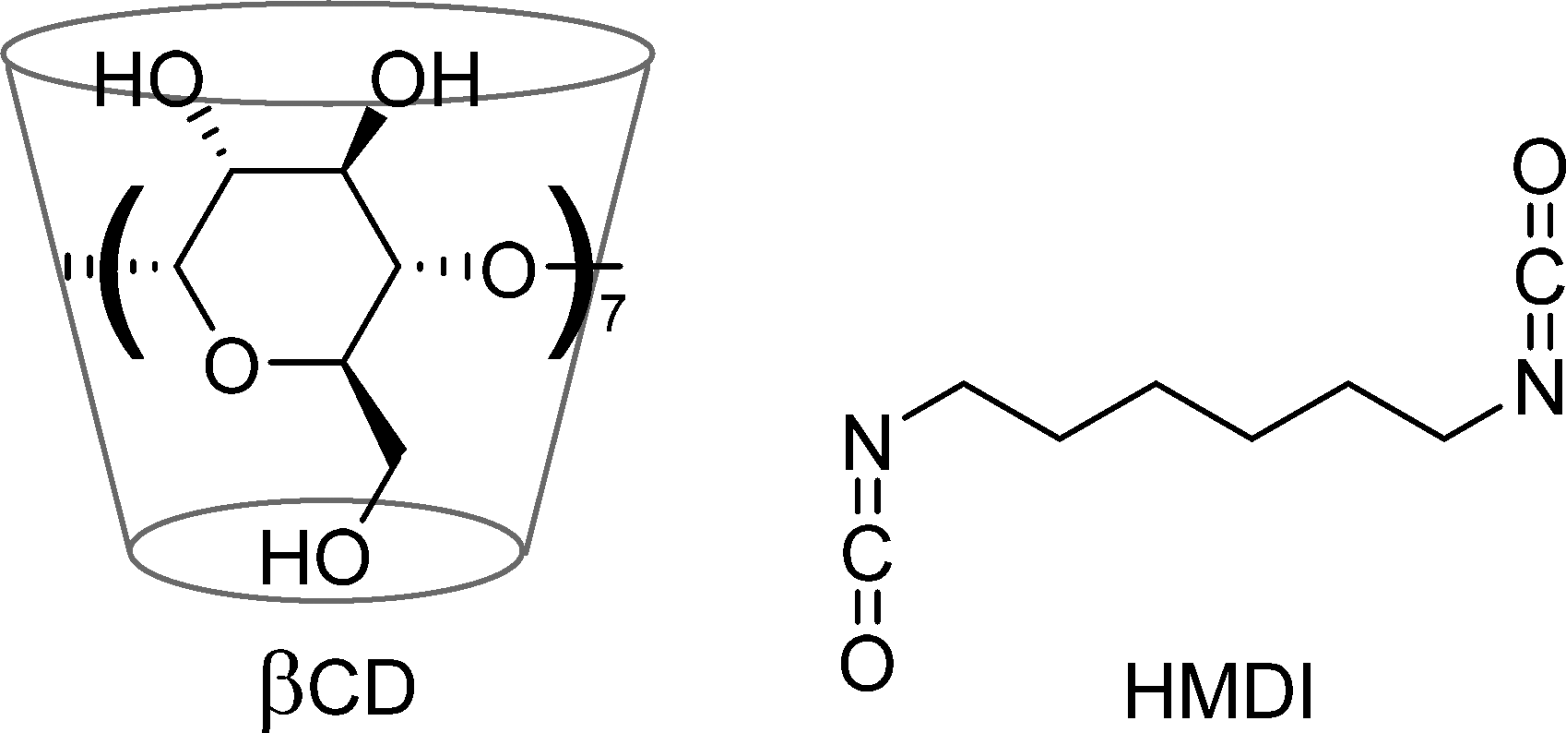
Structures of βCD and HMDI.

Due to the presence of channels and sinks in the structure of NSs, we reasoned that the concept of connectivity developed in ecology and soil science might also be suitable for the description of these materials. In order to develop a different methodology for assessing their texture features, in the present work we performed an FFC-NMR study of three NS materials (referred to as NS1, NS2, and NS3) obtained by reacting β-cyclodextrin with hexamethylene-diisocyanate (HMDI, Figure 1).

Different mole-to-mole ratios (i.e., 1:4, 1:2 and 1:1, respectively) were applied, in order to achieve a different extent of reticulation. The reaction between the −OH groups of βCD and the −N=C=O groups of HMDI results in the formation of urethane units. The relaxometric behavior of the nanosponges obtained was studied for both dry and wet samples, in order to consider the dynamics of water molecules into the nanochannels subjected to possible swelling.

## Experimental Section

### Materials and Instrumentation

All the reagents and solvents needed were used as purchased (Sigma-Aldrich, Cyclolab), without further purification. Anhydrous β-cyclodextrin was obtained by drying the commercial hydrated product in vacuo over phosphorus pentoxide at 90 °C overnight.

Spectrophotometric determinations of *p*-nitrophenol concentration (for obtaining absorption isotherms) were carried out with a Beckmann Coulter DU 800 apparatus. ATR-FTIR spectra were recorded on a PerkinElmer SPECTRUM TWO instrument. The N_2_ absorption–desorption isotherms were registered at 77 K using a Quantachrome Nova 2200 multistation high speed gas sorption analyzer. Thermogravimetric analyses were performed on a Q5000 IR apparatus (TA Instruments) under nitrogen flow (25 cm^3^ min^–1^).

### Synthesis and FTIR Characterization

Preparation of materials NS1, NS2, and NS3 was accomplished according to literature reports.^35^

#### NS1

Anhydrous βCD (568 mg, 5 × 10^–4^ mol) was mixed in a glass vial with a solution prepared by dissolving 320 μL of HMDI (336 mg, 2 × 10^–3^ mol) in 400 μL of dry DMSO. The system was mechanically stirred with a tiny steel rod to ensure effective mixing and then was kept still at 60 °C for 18 h. The hard reaction crude was coarsely crunched and suspended into 40 mL of distilled water; the suspension was sonicated for ca. 10 min, and the solid residue was finally recovered by centrifugation (5500 rpm for 10 min). The residue was then suspended in 40 mL of methanol, sonicated, and centrifuged as described above. The same washing procedure was iterated with another portion of methanol (40 mL) and then with diethyl ether (40 mL). The solid was finally recovered by filtration, finely crunched, passed through a 150 μm sieve, and dried overnight, by being kept in vacuo at 60 °C over phosphorus pentoxide. Yield 885 mg.

#### NS2

The same procedure as for NS1 was followed, starting from 568 mg (5 × 10^–4^ mol) of βCD and 160 μL (168 mg, 1 × 10^–3^ mol) of HMDI. Yield 721 mg.

#### NS3

The same procedure as for NS1 was followed, starting from 568 mg (5 × 10^–4^ mol) of βCD and 80 μL (84 mg 5 × 10^–4^ mol) of HMDI. Yield 455 mg.

In general, the accomplishment of the reticulation process was immediately verified by the mechanical hardness and lack of solubility of the reaction products, which were recovered almost quantitatively in the cases of NS1 and NS2. Conversely, NS3 was partly soluble in water, thereby providing a lesser reaction yield (ca. 70%). The latter observation can be justified by the insufficient amount of HMDI, which prevented a full reticulation.

**Figure 2:**
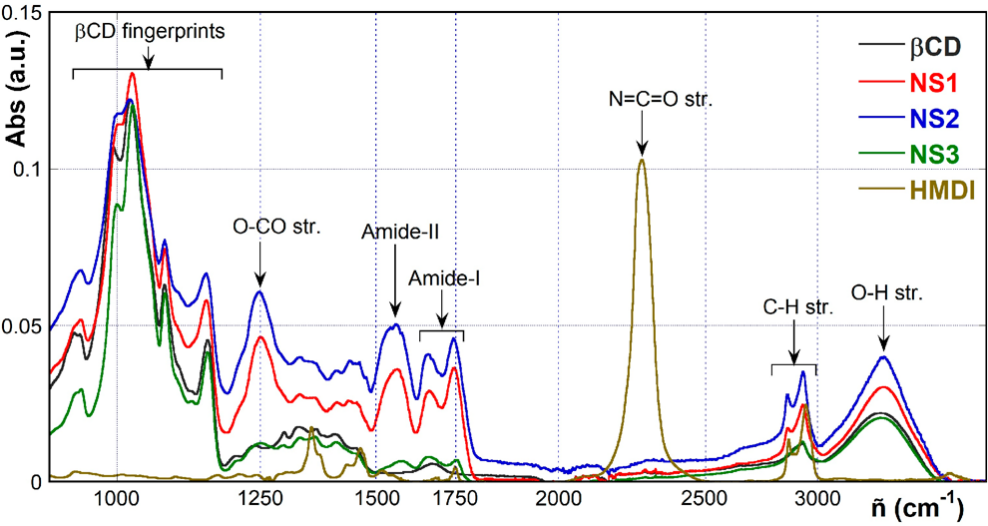
ATR-FTIR spectra of βCD and HMDI and materials NS1, NS2, NS3.

The formation of the polymeric network was confirmed by ATR-FTIR analysis (spectra are synoptically shown in Figure 2). The main features in the spectrum of the starting βCD are the large O–H str (stretching) band centered at 3304 cm^–1^, followed by a tiny C–H str signal at 2918 cm^–1^ and by the typical βCD fingerprints system (various C–O str) in the range ∼1200–900 cm^–1^. On the other hand, the spectrum of HMDI shows a system of two signals at 2940 and 2863 cm^–1^ (asymmetric and symmetric str of the methylene units) and an intense band at 2276 cm^–1^ (−N=C=O str). On passing to nanosponge materials, NS1 and NS2 spectra show the following main features: (i) two C–H str signals at 2928 and 2857 cm^–1^, accounting for the presence of methylene groups of the linker bridges; (ii) a system of three signals at 1694, 1630 (amide-I-like carbonyl str), and 1548 cm^–1^ (amide-II-like N–H bend), accounting for the presence of the urethane groups formed during the reticulation; and (iii) a strong signal at 1251 cm^–1^, conceivably due to the O-carbonyl str of the urethane groups. The same signals, though much weaker, can also be identified in the spectrum of NS3. Finally, all the NSs show the βCD fingerprints at ∼1200–900 cm^–1^. Thus, the actual accomplishment of the reticulation reaction (and the fact that the materials are not mere physical mixtures of the reactants) is positively assessed by the simultaneous presence of signals that can be traced back to the βCD and the hexamethylene chain backbones, and to the newly formed urethane functional groups as well, together with the absence of any signal relevant to unreacted isocyanate groups.

### Relaxometry

Relaxometric experiments were performed on a Stelar Spinmaster FFC 2000 relaxometer (Stelar s.r.l., Mede, PV-Italy) at the constant temperature of 25 °C. The proton spins were polarized at a polarization field (*B*_POL_) corresponding to a proton Larmor frequency (ω_L_) of 10 MHz for a period of polarization corresponding to about 5 times the *T*_1_ estimated at this frequency. After each *B*_POL_ application, the magnetic field intensity (indicated as *B*_RLX_) was systematically changed through the proton Larmor frequency ω_L_ range 0.015–35 MHz. The period τ, during which *B*_RLX_ was applied, was varied on 32 logarithmic spaced time sets, each of them adjusted at every relaxation field in order to optimize the sampling of the decay/recovery curves. FIDs were recorded following a single ^1^H 90° pulse of 5.5 μs applied at an acquisition field corresponding to the proton Larmor frequency of 7.20 MHz. A time domain of 100 μs sampled with 1000 points was applied. The field-switching time was 3 ms, while the spectrometer dead time was 15 μs. For all experiments, a recycle delay of 1 s was used. The nonpolarized FFC sequence was applied when the relaxation magnetic fields were in the range of the proton Larmor frequencies between 35 and 9 MHz. A polarized FFC sequence was applied in the proton Larmor frequencies *B*_RLX_ range 9–0.015 MHz.

## Results and Discussion

### Porosimetry

Porosimetric determinations (i.e., N_2_ absorption isotherms) performed on the fully insoluble materials NS1 and NS2 afforded, as expected, very poor results, partly beyond the lower sensitivity limit of the applied instrumental apparatus. In particular, by means of the BET analysis, an *S* value of 0.134 m^2^ g^–1^ for NS2 only was found. The use of the BJH analysis resulted in *S* values of 0.115 and 0.280 m^2^ g^–1^ for NS1 and NS2, respectively. This trend follows the reticulation degree expected on the grounds of the amount of reticulating agent used in the syntheses. A *V* value of 0.001 cm^3^ g^–1^ was found for both NS1 and NS2, with an average pore diameter of 7.1 and 3.8 nm for the two systems, respectively. The latter finding does not appear, consistent with the expected reticulation degree. On the whole, these results agree with those reported elsewhere,^35^ thereby confirming that N_2_ absorption is not a suitable method for the measurement of nanosponge textures. The reasons of such a failure are unclear, and currently an object of debate.^35^ Probably, the assumptions on which the BET/BJH analyses rely cannot be properly applied in the case of our materials. In particular, the equivalence of the binding sites onto the surface of the nanopores cannot be taken for granted on a microscopic scale; moreover, the system can hardly be modeled as a network of cylindrical pores, as provided by theory.

We also evaluated the specific surface areas and volumes of NS1 and NS2 by means of the *p*-nitrophenol absorption method. Absorption isotherms were studied at 25 °C in aqueous acetate buffer at pH 4.4. Data were subjected to regression analysis by means of the Sips equation in the form *q*_e_ = *q*_max_(*Kc*_eq_)*^n^*/[1 + (*Kc*_eq_)*^n^*], where *q*_e_ and *q*_max_ are the amount of guest absorbed at equilibrium and the maximum capacity of the material (mol g^–1^), respectively; *c*_eq_ is the concentration of the guest at equilibrium; *K* is the apparent equilibrium constant; and *n* is an empirical coefficient. We found the following for NS1: *q*_max_ = (7.8 ± 0.6) × 10^–4^ mol g^–1^, *K* = (2.1 ± 0.2) × 10^3^ M^–1^, *n* = 1.36 ± 0.08. For NS2, *q*_max_ = (7.9 ± 0.9) × 10^–4^ mol g^–1^, *K* = (1.74 ± 0.06) × 10^3^ M^–1^, and *n* = 1.21 ± 0.09. As a consequence, very similar apparent *S* (250 ± 30 m^2^ g^–1^) and *V* (0.043 ± 0.004 cm^3^ g^–1^) values can be estimated for both materials. These results are comparable with those reported by Wilson et al.^35^ for a material with the same composition as NS2 (i.e., *q*_max_ = 11.5 × 10^–4^ mol g^–1^, *S* = 364 m^2^ g^–1^). The discrepancy between the *q*_max_ values is likely due to the presence of the buffer, according to the well-known effect of electrolytes on the binding equilibria of free cyclodextrins in solution.^64−66^

The previous results can be interestingly compared with the composition of NS1 and NS2, which contain 5.5 × 10^–4^ and 6.8 × 10^–4^ mol g^–1^ of βCD, respectively. In general, *p*-substituted nitrobenzene derivatives form in solution only 1:1 complexes both with native and chemically modified βCDs;^67−70^ however, due to the smaller volume of these guests as compared to the host cavity, a simultaneous dynamic coinclusion of solvent molecules occurs.^70^ Moreover, evidence has been reported (2D-LFSE solid-state NMR) that, in the case of the inclusion of a *p*-nitroaniline derivative in a polyamino-cyclodextrin NS, the guest specifically occupies the cyclodextrin cavities and does not reside in the nanochannels.^18^ Now, the *q*_max_ values found experimentally suggest that most of the *p*-nitrophenol guest should be included into the host cavities, whereas only a minor amount (i.e., ca. 30% for NS1, ca. 14% for NS2) may reside into the nanochannels. This implies that the channel surface, which represents the interspace between the cyclodextrin units, is not adequately sampled. Furthermore, taking into account the intrinsic volume of the βCD cavity (i.e., 0.262 nm^3^, which corresponds to 15.77 cm^3^ mol^–1^ or 0.14 cm^3^ g^–1^), from the composition of NS1 and NS2, one can calculate that, even neglecting the contribution from nanochannels, minimum *V* values as large as 0.087 and 0.107 cm^3^ g^–1^, respectively, should be expected. The latter values are much larger than the experimental results. Thus, we can conclude that the use of the *p*-nitrophenol as a probe actually leads to underestimating both *S* and *V* values. Therefore, even the reliability of this approach appears seriously questionable.

### FFC-NMR Relaxometry

**Figure 3:**
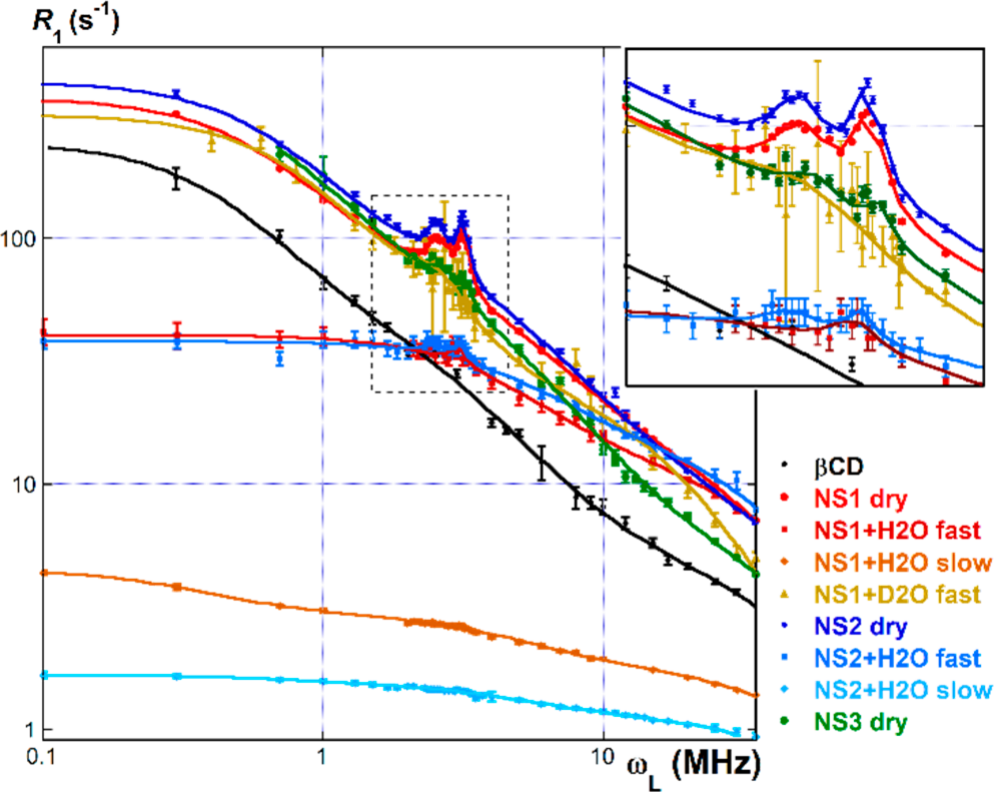
NMR dispersion curves (*R*_1_ vs ω_L_).

Relaxometry experiments performed here aimed at studying the variations of the longitudinal relaxation rates (*R*_1_) in the magnetic field range 0.1–35 MHz. We first investigated the dry NS1–NS3 samples; anhydrous βCD was also analyzed for useful comparison. Then, we considered the two fully insoluble materials, NS1 and NS2, after equilibration with water, added in a 1:2 w/w amount (the relevant samples are indicated hereinafter as NS1 + H_2_O and NS2 + H_2_O). Hence, information on the dynamics at the solid–liquid interface was achieved. Finally, the dynamics of the skeletal ^1^H nuclei was also studied by equilibrating NS1 with D_2_O (1:2.5 w/w, sample NS1 + D_2_O). As a general remark, for the dry samples (βCD, NS1, NS2, NS3) ^1^H relaxation followed a simple first-order exponential trend. Conversely, relaxation of the wet samples (i.e., NS1 + H_2_O, NS1 + D_2_O, NS2 + H_2_O) showed a more complex kinetic profile, which was suitably modeled as the sum of two distinct first-order processes: a “fast” and a “slow” one. This peculiar behavior is mirrored by the inverse-Laplace transform analysis of the relaxation kinetic curves (see later). For the sake of completeness, we must mention here that for the NS1 + D_2_O sample the slow component was affected by very large errors, thereby showing a very scattered trend with respect to the proton Larmor frequency (ω_L_); therefore, it was not further analyzed. NMR dispersion curves (*R*_1_ vs ω_L_) for all the samples are synoptically shown in Figure 3 (the complete data set is reported in the Supporting Information, Table S1).

Interestingly, most of the dispersion curves show the presence of the typical dips located at ω_L_ ca. 2.7 and 3.1 MHz, due to the quadrupolar coupling effect with the urethane N atoms (inset in Figure 3). Dips are very detectable not only in the curves relevant to dry materials NS1, NS2, and NS3, but also for the fast components of the NS1 + H_2_O and NS2 + H_2_O samples. The latter finding suggests the occurrence of strong long-range N–H interactions even under conditions that are able to favor proton exchange. Conversely, dips are absent in the dispersion curves relevant to the slow component of the wet samples, to the fast component of NS1 + D_2_O and, obviously, to dry βCD.

NMR dispersion curves were subjected to regression analysis according to Halle’s approach,^71−73^ as the sum of two or three “stretched” components, to which two additional Lorentzian-like contributions were added in order to account for the simultaneous occurrence of the quadrupolar dips (when needed):
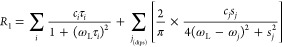
1

From the fitting parameters (reported in the Supporting Information, Table S2), the relevant correlation times (τ_c_’s) for each curve were calculated (Table 1), according to the relationship
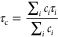
2

**Table 1:** Correlation Times (τ_c_’s)

sample	τ_c_ (ns)	sample	τ_c_ (ns)
βCD dry	600 ± 180		
NS1 dry	430 ± 40	NS2 dry	450 ± 50
NS1 + H_2_O fast	68 ± 5	NS2 + H_2_O fast	58 ± 4
NS1 + H_2_O slow	37 ± 4	NS2 + H_2_O slow	20 ± 4
NS1 + D_2_O fast	500 ± 90	NS3 dry	580 ± 240

For molecular compounds, τ_c_ is interpreted as the average time needed for a molecule to rotate one radian or to move within a distance equal to its length. Therefore, it can be considered as a suitable measure for structural mobility at a microscopic scale. Dry samples showed similar correlation times, within the limits of experimental uncertainties. By contrast, wet samples NS1 + H_2_O and NS2 + H_2_O showed much smaller τ_c_ values for the fast component, and even smaller values for the slow one. Both correlation times for NS1 + H_2_O are significantly larger than those for NS2 + H_2_O. Noticeably, sample NS1 + D_2_O shows a comparable τ_c_ value with respect to the relevant dry material. These observations suggest that correlation times for samples with H_2_O mainly keep into account the relaxometric behavior of water molecules. However, we can distinguish at least two different populations having a significantly different mobility. Trends for τ_c_ values are consistent with the different reticulation degree expected for the materials. In fact, NS1 is supposed to present significantly narrower channels than NS2, having been prepared with a larger amount of reticulating agent. Therefore, we may reasonably relate the fast component (longer τ_c_) of the relaxation kinetics to H_2_O molecules which are tightly bound to the nanosponge network, thereby forming its immediate hydration shell/layer. This idea is supported by the residual presence of the quadrupolar dips observed in the relevant dispersion curve. Conversely, the slow component (shorter τ_c_) may be associated with loosely bound molecules flowing within the lumen of the channels. For this purpose, for the sake of information completeness, it is worth mentioning here that dry materials retain a small percentage of water, ca. ∼5–7% (by TGA analysis). On the grounds of trivial stoichiometric calculations, this on average corresponds to 7 ± 2 water molecules per βCD unit. It is quite reasonable to assume that these water molecules are very tightly bound to the structure either via hydrogen bonding, or as small clusters placed inside the βCD cavities. Thus, keeping into account the theoretical molecular formulas (namely, [(C_42_H_70_O_35_)·(C_8_H_12_N_2_O_2_)_4_]*_n_* for NS1, [(C_42_H_70_O_35_)·(C_8_H_12_N_2_O_2_)_2_]*_n_* for NS2), it can be estimated that nearly 75% of the H atoms present in the samples are stably bound to C atoms, whereas the remaining 25% are “exchangeable protons” bound to O or N atoms. Differently, for both the wet samples NS1 + H_2_O and NS2 + H_2_O, it can be calculated that the exchangeable protons correspond to ca. 79% of the total H atoms population.

Close inspection of the dispersion curves tells a more articulated story. Seen on a logarithmic scale, curves for dry NS1 and NS2 appear to be almost superimposable at large ω_L_ values and only slightly diverging at low ω_L_. This suggests that relaxation mechanisms are very similar in the two cases. However, relaxation for anhydrous βCD is much slower in the whole ω_L_ range, clearly indicating that lack of reticulation favors molecular motions on a microscopic scale. The curves for dry NS3 and NS1 + D_2_O are in an intermediate position between βCD and dry NS1 at large ω_L_, while they approach the latter ones at low ω_L_ values. This suggests an intermediate structural mobility. In the former case, this can be justified with the incomplete reticulation due to the low combination ratio between βCD and HMDI (i.e., 1:1 mol/mol), whereas, in the latter case, it positively indicates a swelling occurrence. On passing to wet samples, curves for the fast component of NS1 + H_2_O and NS2 + H_2_O approach those for the corresponding dry materials only at the largest ω_L_ values, but neatly diverge in the region at low ω_L_, where the relaxation rates for both samples are very similar and almost independent of the Larmor frequency. This peculiar behavior suggests that different sections of the dispersion curves account for different aspects of the microscopic dynamism of the samples. In particular, curve trends suggest that the relaxometric response appears more sensitive to not-exchangeable C-bound protons and to tightly bound water molecules of the hydration layer at the larger ω_L_ values. Conversely, larger sensitivity to less tight, easily exchangeable water molecules seems to exist at the lower ω_L_ values. Finally, the quadrupolar dips should specifically account for the behavior of protons bound to the urethane N atoms.

**Figure 4:**
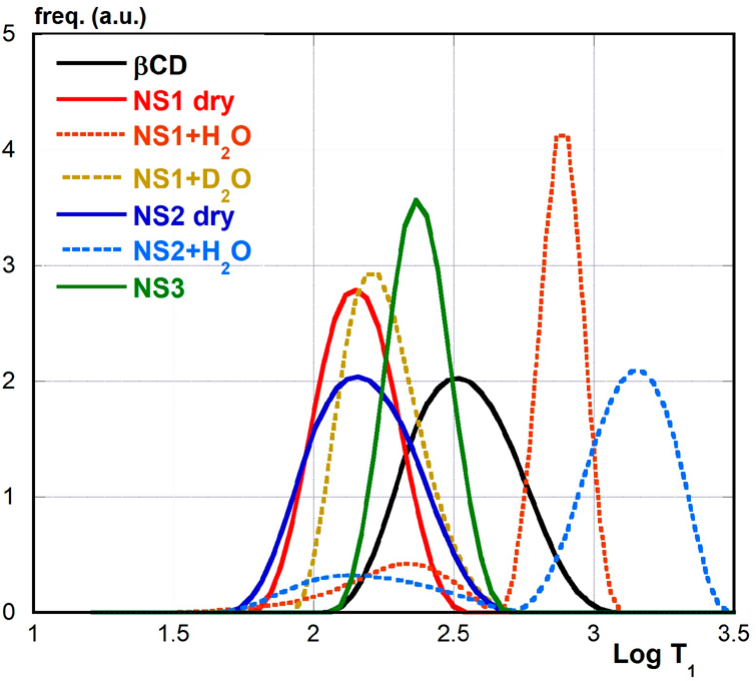
Normalized inverse-Laplace transforms (UPEN) at 35 MHz.

In order to clarify and support these hypotheses, we considered the inverse-Laplace transform curves of the relaxation kinetics data at five different ω_L_ values (namely, 35, 10, 3, 1, and 0.3 MHz), obtained by means of the UPEN algorithm. Normalized transform curves (a representative example is shown in Figure 4) can be subjected to regression analysis as skewed log-normal distributions, the maximum and the full width at half-height (fwhh) values of which were considered (Table 2, the complete data set is reported in the Supporting Information, Table S3 and Figures S2–S6).

**Table 2:** Parameters for the Inverse-Laplace Transforms (UPEN)

sample	ω_L_ (MHz)	max (ns)	fwhm (ns)	pop. (%)	sample	ω_L_ (MHz)	max (ns)	fwhm (ns)	pop. (%)
βCD	35	303	141	100	NS1 dry	35	142	47	100
	10	122	49	100		10	46	26	100
	3	36.2	12.1	100		3	10.7	2.8	100
	1	13.3	8.3	100		1	6.6	2.1	100
	0.3	5.1	1.8	100		0.3	2.8	0.7	100
NS2 dry	35	145	67	100	NS3 dry	35	234	62	100
	10	43	24	100		10	76	33	100
	3	9.5	3.8	100		3	16.3	7.3	100
	1	5.9	2.3	100		1	4.6	2.9	100
	0.3	2.5	1.2	100					
NS1 + H_2_O	35	770	319	80	NS2 + H_2_O	35	1442	1023	81
		231	205	20			129	117	19
	10	526	352	82		10	906	1164	83
		66	106	18			44	40	17
	3	394	461	84		3	860	1175	82
		19.0	22.6	16			11.2	16.0	18
	1	392	382	85		1	793	888	83
		13.2	12.9	15			9.9	11.7	17
	0.3	314	328	91		0.3	715	1003	86
		9.8	11.9	9			5.8	13.5	14
NS1 + D_2_O	35	160	124	100					
	10	734	714	37					
		52	52	63					
	3	14.3	32	100					
	1	210	226	9					
		10.6	14.6	91					
	0.3	179	274	21					
		3.0	7.1	79					

As long as dry samples are concerned, all inverse-Laplace transforms afford a unimodal distribution at any Larmor frequency, according to the observed first-order relaxation kinetics. Consistently with the discussion of dispersion curves reported hereinabove, the distribution maxima increase along the series NS2 ≈ NS1 < NS3 < βCD. This trend fairly agrees with the reticulation extent of the materials, but for the fact that values for NS1 are slightly larger than for NS2. The latter apparent anomaly might be justified with the much larger presence in NS1 of the hexamethylene linker chains, which benefit from a moderate conformational flexibility. Conversely, transforms for wet samples show bimodal distributions. Their major component is centered at larger *T*_1_ values as compared to those found for dry materials, whereas the minor component is centered at comparable *T*_1_ values. This finding, in turn, is consistent with the occurrence of the complex relaxation kinetics observed in these cases. More in detail, the two components correspond to the slow and fast counterparts of the relaxation kinetics, respectively. As long as the samples with H_2_O are concerned, the two distribution maxima for NS1 occur at much lower *T*_1_ values in comparison to NS2, consistent with its more extensive reticulation. On the other hand, maxima for the fast component occur at significantly larger values with respect to dry materials, accounting for larger molecular motions, which can be attributed to both the skeletal ^1^H atoms and the hydration shell water molecules. The distribution maximum relevant to the fast component for the NS1 + D_2_O sample is in an intermediate position between the ones for dry NS1 and NS1 + H_2_O. The latter detail confirms the effect of swelling in increasing molecular motions for the skeletal H atoms in the wet sample. Finally, the relative populations of the two components significantly change on varying the Larmor frequency. In particular, for NS1 + H_2_O the fast component passes from a 20% population at 35 MHz down to 9% at 0.3 MHz, whereas for NS2 + H_2_O, the fast component passes from 19% down to 14% population in the same ω_L_ range. The latter observations strongly support the idea that the sensitivity of the relaxometric response to water molecules tightly interacting with the nanosponge framework decreases on decreasing the Larmor frequency.

### Texture Features and Connectivity Indexes

At this point the question arises as to how the relaxometric behavior observed may enable researchers to gain information on the texture properties. According to theory,^54−56,74^ for wet micro- or nanoporous materials the relaxation rates *R*_1_ can be related to the fraction of water molecules interacting with the pore surface (*f*_S_) by the equation
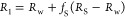
3where *R*_w_ is the relaxation rate of bulk water (ca. 0.33 s^–1^,^55^ almost independent of ω_L_ in the frequency range analyzed in this study) and *R*_s_ is the intrinsic relaxation rate for “surface” water. Hence, *R*_1_ can lead back to the porosimetric parameters of the material according to the relationships
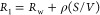
4
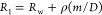
5
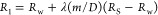
6where ρ is a parameter defined as the “surface relaxivity” (in the case of clay materials or natural soils, for instance, ρ values have been reported^55,56,74^ in the range ca. ∼5–60 μm s^–1^), λ represents the thickness of the hydration shell of pore walls (usually set as large as 0.3 nm), and *m* is a geometry parameter, the value of which is a function of pore shape (*m* = 4 for cylindrical pores, 6 for spherical ones). By combining eqs 3–6, the two relationships *D* = λ(*m*/*f*_S_) and *f*_S_ = λ(*S*/*V*) can be deduced with few trivial passages.

According to the previous discussion, in our case we can assume that the fast and slow components of the relaxation kinetics for wet samples adequately describe the behavior of surface and pore water molecules, respectively. Consequently, we deduce
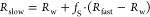
7and then:
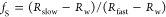
8

**Figure 5:**
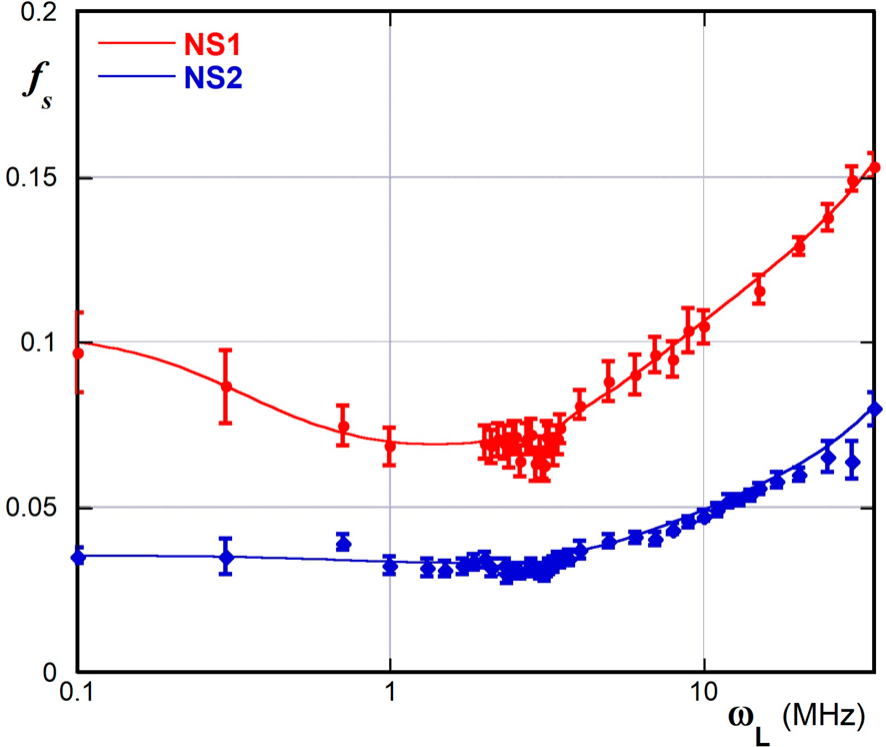
Apparent dependence of *f*_S_ on ω_L_ for NS1 and NS2.

Interestingly, the apparent values of *f*_S_ calculated from our data vary as a function of ω_L_ (Figure 5, see data in the Supporting Information, Table S4), passing through a minimum located between 1 and 2 MHz. This implies that even the apparent thickness λ of the hydration shell of the pore surface depends on ω_L_ as well, according to the previous relationship *f*_S_ = λ(*S*/*V*). In fact, the ratio *S*/*V* must be a constant as an intrinsic feature of the material.

We may reason that the value of 0.3 nm assumed for λ in the case of clays and soils is conceivably unsuitable for nanosponges. In fact, water molecules can form clusters into the βCD cavities (0.65 nm). It is reasonable to assume that a similar behavior may also be shown by water molecules constrained into channels of similar width. Moreover, the secondary rim of cyclodextrins is able to exert a highly ordering effect on the water molecules located in its immediate surroundings, in such a way as to form a so-called “expanded hydrophobic sphere”.^75−77^ The presence of this peculiar arrangement of water molecules is able to significantly affect the thermodynamics of binding of long-chain guests in solution. In particular, there is positive evidence that the size of the “expanded hydrophobic sphere” is roughly as large as an extended propylene diamine chain (i.e., ca. 0.5 nm).^78^ Finally, the hydrophobic hexamethylene chains may induce an extensive clusterization of water molecules, due to the hydrophobic effect. This, in turn, affects the long-range solvent organization in the nearby. On the whole, consistent with the discussion of the *R*_1_ vs ω_L_ curves reported hereinabove, our results can be rationalized assuming that the relaxometric response is able to sample at the largest ω_L_ values a long-range structuration of the pore solvent shell, that is somehow less perceived at the lowest ω_L_ values.

From eq 8, the average pore diameters of the materials may be related to relaxation rates according to the following expressions:
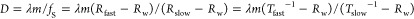
9Unfortunately, on the basis of the previous discussion, eq 9 cannot be applied in our case, because neither λ nor *m* values can be satisfactorily set. In fact, λ is ω_L_-dependent; moreover, it is hardly possible to provide a reasonable value for the pore-shape-dependent *m* parameter, because of both the disordered NS microscopic structure and the unpredictable swelling effects. Nevertheless, it is interesting to notice that (as can be easily verified from the data in Table S4) the ratio between the *f*_S_ values for NS1 and NS2 is independent of ω_L_. More in detail, we found *f*_S,NS1_/*f*_S,NS2_ = 2.2 ± 0.3, which hence provides a reasonable estimation of the ratio between the average pore diameters for the two materials.

Furthermore, the existence of a distribution of relaxation times, mirroring pore size distribution, must be considered. Indeed, the normalized inverse-Laplace transforms provide a frequency distribution function (*P*(*T*_1_)) for the *T*_1_ values, so that we can define an average relaxation time ⟨*T*_1_⟩ as
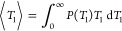
10In order to apply eq 9, average values for *T*_fast_ and *T*_slow_ should be first calculated according to eq 10, by considering independently the normalized fitting expressions for the fast and the slow components in place of *P*(*T*_1_). Noticeably, because *D* can be expressed as a function of *T*_fast_ and *T*_slow_ by eq 9, the normalized frequency distribution *P*(*T*_1_) for the isolated slow component may be transformed in a frequency distribution function for pore diameters (*P*′(*D*)) by a trivial variable exchange, i.e.
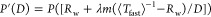
11Then, the average pore size could be calculated as
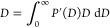
12Once again, lack of reliable values for λ and *m* forbids defining the function *P*′(*D*); so, even calculation of *D* according to eq 12 is not possible.

The intrinsic difficulties in establishing a satisfactory method to evaluate the proper textural parameters, which are apparent from the whole of the previous discussions, moved us to explore some possible alternative approaches. In particular, we reasoned that the features of soils are somehow comparable to those of nanosponges and may constitute a suitable study model. As we mentioned in the Introduction, soil properties have been adequately described, on the basis of the relaxometric responses, by introducing the concept of “connectivity”, intended both in a “structural” (i.e., the through-space interconnection of the pore network) and in a “functional” (i.e., the ability to mediate physical, chemical, and biological phenomena) sense. It is worth stressing that the key point here is the fact that “connectivity” provides an elegant and viable way to describe the functional role of water in mediating the transport phenomena through the channel network, and how this affects the overall interaction/exchange abilities of the porous system. It is immediately apparent that such a role is perfectly mirrored in NS materials. Accordingly, two connectivity indexes have been defined,^62,63^ namely, a “functional connectivity index” (FCI) and a “structural connectivity index” (SCI), able to provide a suitable quantitative assessment of these properties. More in detail, starting from the probability function *P*(*T*_1_) defined in eq 10, two arbitrary reference *T*_1_ values (*T*_A_ and *T*_B_) are first set according to the conditions
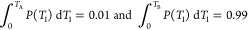
13in such a way that the interval *T*_A_–*T*_B_ comprises the most significant part (98%) of the *T*_1_ values distribution, accounting for water molecules’ mobility. Then, the aforementioned connectivity indexes are simply defined as FCI = *T*_B_/*T*_A_ and SCI = *T*_B_ – *T*_A_.

In order to apply these ideas in our case, we preliminarily chose to adopt a slightly more restrictive significance criterion, keeping into account only 95% of the overall ^1^H population. Therefore, we defined *T*_A_ and *T*_B_ by fixing the upper integration limits in eqs 13 at 0.025 and 0.975, respectively. Then, considering that *f*_S_ linearly depends on the average pore diameter *D* by a λ*m* factor (and to the *S*/*V* ratio as well, eqs 3–6), in analogy with eq 9, we decided to define a further “pore connectivity index” (PCI) according to the relationship
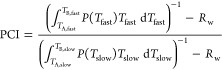
14Thus, PCI is in fact directly related to *D*, through the parameters λ and *m*. The values of FCI, SCI, and PCI calculated from the relaxometric data relevant to the wet samples are collected in Table 3.

**Table 3:** FCI, SCI, and PCI Values for NS1 and NS2

	NS1			NS2		
ω_L_ (MHz)	FCI	SCI	PCI	FCI	SCI	PCI
35	1.98	547	4.3	3.30	1713	13.1
10	2.98	652	6.2	7.05	2750	28.0
3	6.36	953	18.2	7.15	2132	69.3
1	5.25	595	27.7	5.98	1708	87.5
0.3	5.58	565	26.0	8.74	2088	46.4

At any Larmor frequency, the values of the three indexes are larger for NS2 than for NS1. This is perfectly consistent with the different reticulation extent. In agreement with the findings of previous works on soils,^62^ neither FCI nor SCI values show regular variations as a function of ω_L_. However, we can notice that FCI tends to decrease on increasing ω_L_, whereas SCI might be considered roughly constant. Differently, PCI values for both materials pass through a maximum, centered at 1 MHz. Again, the ratio between the PCI values for the two materials does not depend on ω_L_, within the reasonable statistical variations (PCI_NS2_/PCI_NS1_ = 3.2 ± 0.6). Therefore, PCI can be considered as a measure of the different mobilities of water molecules within the nanochannels of the two different materials. On the whole, these findings suggest that PCI can be considered an excellent alternative parameter (with respect to the textural *D*, *S*, and *V* values), providing a satisfactory assessment of the textural features of nanosponges from a functional standpoint. In particular, PCI has the advantage to bypass the intrinsic difficulties deriving from the poor definition of the λ and *m*, the values of which critically depend on the highly disordered and swellable microscopic NS structure. Moreover, in comparison with the previous FCI and SCI indexes, PCI benefits from the fact that its trends depend on ω_L_ in a way that appears easier to be rationalized.

As a final remark, it is worth stressing that, from a general reconsideration of the entire topic based on the analysis of the whole of the experimental results, the definition of the ordinary textural properties (*D*, *S*, *V*) appears unsatisfactory and elusive in the case of nanosponges. Indeed, the very fact that the structure of these materials may undergo fair swelling in the presence of an aqueous medium, with the consequent effect on the overall structure, causes unpredictable variations of pore sizes; for the same reason, even the concept itself of surface seems somehow to fade away at such a microscopic scale. Therefore, it makes much more sense, in our opinion, to define a porosity parameter in functional terms. This requirement seems adequately fulfilled by the connectivity indexes such as PCI.

## Conclusions

This study reports for the first time the application of fast-field-cycling NMR relaxometry in order to gain information on the textural features of nanosponges. In particular, the study of wet samples revealed a complex behavior, which was attributed to the existence of two different water molecule populations, having significantly different relaxation rates and molecular mobility. This, in turn, led back to the pore size distribution in the materials. Moreover, inspired by the results from soil science, we adapted to nanosponges the concepts of connectivity indexes. From the inverse-Laplace *T*_1_ distributions functions, a new “pore connectivity index” (PCI) was suitably defined (eq 14), the values of which are positively correlated to the reticulation degree of the synthesized NSs. Although the PCI index is positively related to the pore size distribution, it provides more than a mere assessment of structural features but rather accounts for the functional properties of the nanosponge materials related to the mobility of water throughout the pore network. In this sense, it is noteworthy that the PCI definition trespasses the intrinsic conceptual difficulties in assessing the texture features deriving from the unpredictable structural effects of swelling.

Finally, even though it cannot directly provide a quantitative measure of textural parameters, FFC NMR relaxometry proved to offer an easy and viable methodology to assess the functional features of nanosponges. It is undoubtedly more reliable and less time- and material-consuming than ordinary porosimetric methods based on the analysis of absorption isotherms. This, in turn, makes it a valuable tool for rationalizing the supramolecular behavior and abilities of nanosponges. Therefore, it may be helpful for clarifying the molecular mechanisms involved in their controlled absorption and release properties, and, in perspective, for a rational design of tailored systems.

## Supporting Information Available

Short outline of FFC-NMR theory, *R*_1_ vs ω_L_ data, Halle’s regression parameters *R*_1_ vs ω_L_ curves, normalized inverse-Laplace (UPEN) *T*_1_ distribution curves, log-normal regression parameters for inverse-Laplace (UPEN) *T*_1_ distribution curves, and *f*_S_ vs ω_L_ data for NS1 and NS2 (PDF)

## Abbreviations

ATR: attenuated total reflectance
BET: Braunauer–Emmett–Teller theory
BJH: Barrett–Joyner–Halenda theory
FFC: fast-field-cycling
fwhm: full width at half-maximum
