# Supplementary material for: Water Dynamics at the Solid–Liquid Interface to Unveil the Textural Features of Synthetic Nanosponges
Source: J Phys Chem B. 2020 Feb 18;124(9):1847–57. doi: 10.1021/acs.jpcb.9b11935 (PMC7997571; doi:10.1021/acs.jpcb.9b11935)
Supplement: Supplementary file 1 — jp9b11935_si_001.pdf [file jp9b11935_si_001.pdf]

Water Dynamics at the Solid-Liquid Interface to Unveil the Textural Features of Synthetic Nanosponges.

Paolo Lo Meo,*† Federico Mundo,† Samuele Terranova,† Pellegrino Conte,*‡ Delia Chillura Martino.†

† Dept. of Biological, Chemical and Pharmaceutical Sciences and Technologies (STEBICEF), University of Palermo; V.le delle Scienze ed. 17, 90128 Palermo, Italy.

‡ Dept. of Agricoltural, Food and Forest Sciences (SAAF), University of Palermo; V.le delle Scienze ed. 4, 90128 Palermo, Italy.

e-mail: paolo.lomeo@unipa.it; pellegrino.conte@unipa.it.

Supporting Information

i) Short outline of FFC-NMR theory.[1]

Fast field cycling (FFC) NMR relaxometry is the major technique to be applied in studying the behavior of a liquid system on the surface of a porous medium. In particular, as a solid material is added with liquid water, water molecules are subjected to two different types of motions. From the one hand, water molecules stay in the proximity of the solid surface for a short time during which they are subjected to a horizontal diffusion. From the other hand, molecules laying on the surface can leave it and reenters the bulk, while they are replaced by bulk water molecules. The distribution of water motional frequencies depends upon the homogeneity of the surface of the porous medium. Water confined in small-sized pores is more tightly constrained than that freely moving in larger spaces. The distributions of magnetic fields (DMF) due to the motional fluctuations are responsible for the dispersion of the longitudinal (or spin-lattice) relaxation times (T_1) occurring when each frequency in DMF matches the Larmor frequencies (ω_L) of the observed nuclei. In the case of water containing systems, the observed nuclei are the protons in water molecules. Water near the surface can also interact with surficial paramagnetic systems. The resultant modulation of the local dipolar magnetic field generated by paramagnetism additionally contributes to spin-lattice relaxation. The direct relationship between the frequency of the water motion and the ^{1}H Larmor frequency is related to the water dynamics in porous media through the modulation of the intensity of the applied magnetic field. Fast field cycling NMR relaxometry is based on the fast change of the intensity of an applied magnetic field in order to monitor the variations of ^{1}H T_1 values of a dynamic system.

Figure S1 shows the basic FFC NMR experimental design where the typical preparation-evolution-acquisition steps of the basic NMR experiment are replaced by polarization-relaxation-acquisition intervals. As outlined below, a pre-polarized (PP) and a non-polarized (NP) sequence can be recognized. During the first step of the PP sequence, a longitudinal magnetization is generated through the application of a polarization field (B_{POL}) for a limited and fixed period of time (referred to as polarization time, T_{POL}). Afterwards, the magnetic field

is switched to a new one (indicated as relaxation field, B_{RLX}), applied for a period τ during which the magnetization intensity relaxes to reach a new equilibrium condition. Finally, the application of a 1H 90° pulse into an acquisition magnetic field (B_{ACQ}) held for a fixed time makes the magnetization observable and the free induction decay (FID) acquirable. In the NP sequence, B_{POL} is null. The PP sequence is applied when the relaxation field becomes very low in intensity and enhancement of sensitivity is needed for FID achievement. The crossover field between the NP and PP sequences is approximately retrieved when the relaxation field intensity is half of that of the polarization field. The longitudinal relaxation time (T_1) values of the observed nuclei are obtained for every given B_{RLX} through a progressive variation of the τ values.

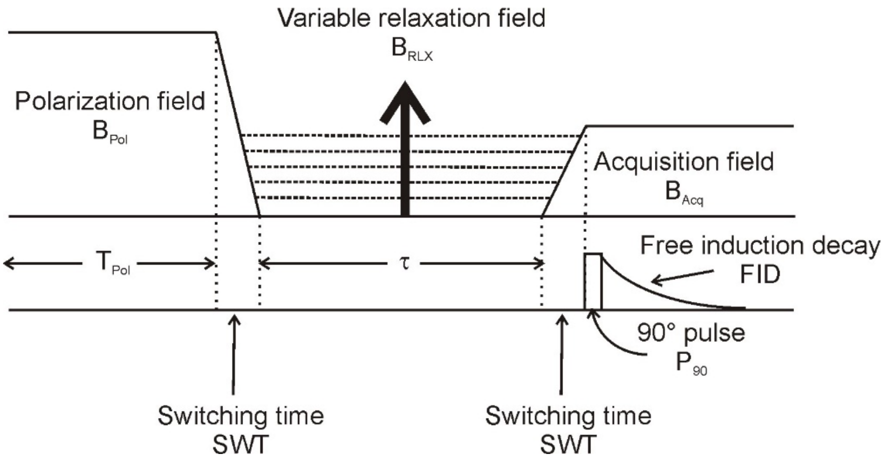

Figure S1. Scheme of the typical sequence used in FFC NMR relaxometry. The non-polarized sequence is obtained when B_{Pol} is null. Conversely, as B_{Pol} is non-null, the pre-polarized sequence is achieved.

The relationship between signal intensity and τ can be modeled as in equation (S1) for the PP sequence and as in equation (S2) for the NP sequence:

$$M(\tau) = a + \sum_{i=1}^{N} b_i \exp\left\{-\frac{\tau}{T_{1i}}\right\} \tag{S1}$$

$$M(\tau) = a + \sum_{i=1}^{N} b_i \left(1 - exp\left\{-\frac{\tau}{T_{1i}}\right\}\right) = \left(a + \sum_{i=1}^{N} b_i\right) - \sum_{i=1}^{N} b_i exp\left\{-\frac{\tau}{T_{1i}}\right\} \tag{S2}$$

In both equations (S1) and (S2), $M(\tau)$ is the magnetization intensity at the selected τ value; a is the offset; b_i is the magnetization intensity at the Boltzmann equilibrium of the i-th component of the molecular motion at each fixed B_{RLX} intensity. Neither equations (S1) and (S2) can correctly represent the continuous distribution of T_1 values when multiphase systems are investigated. When different components of the molecular dynamics in multiphase frames are described by longitudinal relaxation times with values very close to each other, a better representation of their T_1 distributions can be obtained by applying an inverse Laplace transformation. It has the form reported in equation (S3) for the pre-polarized experiments, while it is as in equation (S4) when the non-polarized experiments are done.

$$M(\tau) = \int_{T_{1min}}^{T_{1max}} D(T_1) exp\left[-\left(\frac{\tau}{T_1}\right)\right] d(T_1) + \sigma \quad \text{(S3)}$$

$$M(\tau) = \int_{T_{1min}}^{T_{1max}} D(T_1) \left\{1 - exp\left[-\left(\frac{\tau}{T_1}\right)\right]\right\} d(T_1) + \sigma \quad \text{(S4)}$$

In equations (3) and (4), T_{1min} and T_{1max} are the longitudinal relaxation time limits within which all the T_1 values fall. $D(T_1)$ is the distribution function which has to be determined by solving either equation (S3) or equation (S4); σ is an unknown noise component. The latter makes impossible to find the exact distribution of relaxation times, thereby allowing infinite possible solutions for both the aforementioned equations. However, the most likely distribution of relaxation times can be obtained when some constraints, such as smoothness of the solution and variance of the experimental data, are accounted for. The algorithm used in the present study to retrieve the continuous T_1 distributions the uniform penalty regularization, also referred to as UPEN.

The variations of the longitudinal relaxation rates (R_1=1/T_1) at each B_{RLX} as described above, provide the nuclear magnetic resonance dispersion (NMRD) curves. These latter reflect the spectrum of re-orientational and diffusional molecular dynamics. They are described by Lorentzian functions with the form reported in equation (S5) through which information about correlation functions of the microscopic fluctuations can be achieved.

$$R_1 = \frac{1}{T_1} = \frac{Const \times \tau_C}{1+(\omega_L \tau_C)^2} \quad \text{(S5)}$$

Here, *Const* is a constant parameter, ω_L is the proton Larmor frequency (i.e., the B_{RLX} value), and τ_C is the correlation time (i.e., the time taken for a molecule to rotate one radian or to move a distance of the order of its dimension). The longer the τ_C value, the slower the molecular motions are, thereby revealing restrictions in the motional freedom-degrees of spatially restrained molecular systems. Conversely, as a molecule encompasses faster motions due to higher degrees of freedom in larger spaces, shorter correlation time values are expected.

Equation (S5) is only valid when a non-stretched NMRD curve is obtained. When the NMR dispersion profiles are stretched as a consequence of the complexity of the re-orientational dynamics within the molecular system, the mathematical model developed by Halle and co-workers[2] must be accounted for. It is also referred to as "model-free analysis" and is described as in equation (S6).

$$R_1 = \frac{1}{T_1} = \sum \frac{c_i \tau_{Ci}}{1+(\omega_L \tau_C)^2} \quad \text{(S6)}$$

In equation (S6), ω_L is the proton Larmor frequency, c_i is a fitting parameter, and τ_{Ci} is the ith correlation time of the i-th component of the molecular system. The c_i and τ_{Ci} values are used as in equation (S7) to obtain an average correlation time of the whole system:

$$\langle \tau \rangle = \frac{\sum_i c_i \tau_{Ci}}{\sum_i c_i} \quad \text{(S7)}$$

Bibliography

1. Conte, P., Chapter 10 Environmental Applications of Fast Field-cycling NMR Relaxometry. In *Field-cycling NMR Relaxometry: Instrumentation, Model Theories and Applications,* The Royal Society of Chemistry: 2019; pp 229-254.

2. Halle, B.; Jóhannesson, H.; Venu, K., Model-Free Analysis of Stretched Relaxation Dispersions. *J. Magn. Reson.* **1998,** *135*, 1-13.

ii) Table S1. R_1 vs ω_L data.

βCD		NS1 dry		NS1+H_2O fast		NS1+H_2O slow		NS1+D_2O fast	
ω_L (MHz)	R_1 (s^{-1})	ω_L (MHz)	R_1 (s^{-1})	ω_L (MHz)	R_1 (s^{-1})	ω_L (MHz)	R_1 (s^{-1})	ω_L (MHz)	R_1 (s^{-1})
0.3	175 ± 17	0.3	320 ± 3	0.1	42 ± 5	0.1	4.33 ± 0.09	0.4	250 ± 25
0.7	102 ± 6	0.7	192 ± 2	0.3	40 ± 5	0.3	3.78 ± 0.10	0.6	256 ± 26
1.0	66 ± 3	1.0	142.8 ± 1.4	0.7	38 ± 3	0.7	3.17 ± 0.05	0.8	189 ± 21
1.3	56 ± 3	1.3	120.9 ± 0.9	1.0	40 ± 3	1.0	3.04 ± 0.05	1.3	116 ± 9
1.5	47 ± 3	1.5	110.2 ± 1.0	2.0	34 ± 2	2.0	2.70 ± 0.04	1.5	98 ± 9
1.7	43 ± 2	1.7	98.3 ± 0.7	2.1	36 ± 3	2.1	2.75 ± 0.05	1.8	93 ± 7
2.0	33.5 ± 1.3	2.0	88.3 ± 0.9	2.2	34 ± 2	2.2	2.73 ± 0.05	2	83 ± 7
2.5	34.0 ± 1.1	2.1	88.2 ± 0.5	2.3	35 ± 2	2.3	2.70 ± 0.04	2.1	83 ± 14
3.0	28.0 ± 1.0	2.2	87.8 ± 0.7	2.4	36 ± 3	2.4	2.70 ± 0.04	2.2	77 ± 6
4.0	17.7 ± 0.7	2.3	88.1 ± 0.7	2.5	33 ± 2	2.45	2.72 ± 0.04	2.3	100 ± 10
4.5	16.4 ± 0.5	2.35	93.9 ± 0.9	2.6	37 ± 3	2.5	2.67 ± 0.04	2.4	77 ± 12
5.0	16.0 ± 0.5	2.4	97.1 ± 0.9	2.7	33 ± 2	2.6	2.68 ± 0.04	2.45	62 ± 27
6.0	12 ± 2	2.45	99.3 ± 0.8	2.8	32 ± 2	2.7	2.66 ± 0.04	2.5	77 ± 12
8.0	8.8 ± 0.9	2.5	100.9 ± 0.8	2.9	37 ± 3	2.8	2.62 ± 0.04	2.6	77 ± 6
9.0	8.4 ± 0.5	2.55	101.0 ± 0.6	3.0	35 ± 2	2.9	2.64 ± 0.04	2.7	91 ± 50
10.0	8.1 ± 0.6	2.6	97.5 ± 0.8	3.05	37 ± 3	3.0	2.60 ± 0.04	2.8	63 ± 4
12.0	6.9 ± 0.4	2.7	97.8 ± 1.0	3.1	37 ± 3	3.05	2.65 ± 0.04	2.9	71 ± 20
15.0	5.7 ± 0.3	2.8	92.8 ± 0.9	3.15	32 ± 2	3.1	2.63 ± 0.04	3.0	71 ± 10
17.0	4.9 ± 0.2	2.9	86.3 ± 0.7	3.2	32 ± 2	3.15	2.61 ± 0.03	3.05	67 ± 13
20.0	4.6 ± 0.1	3.0	91.4 ± 0.8	3.3	33 ± 2	3.2	2.56 ± 0.04	3.1	53 ± 6
25.0	4.0 ± 0.1	3.05	98.7 ± 0.7	3.4	31 ± 2	3.3	2.55 ± 0.03	3.15	71 ± 15
30.0	3.6 ± 0.1	3.1	105.3 ± 1.0	3.5	29 ± 2	3.4	2.51 ± 0.04	3.2	56 ± 6
		3.15	107.1 ± 1.0	4.0	25.6 ± 1.3	3.5	2.48 ± 0.04	3.3	53 ± 6
		3.2	100.8 ± 0.9	5.0	22.2 ± 1.5	4.0	2.38 ± 0.03	3.4	56 ± 6
		3.3	91.2 ± 1.0	6.0	20.8 ± 1.3	5.0	2.26 ± 0.03	3.45	53 ± 3
		3.4	73.7 ± 0.5	7.0	18.5 ± 1.0	6.0	2.18 ± 0.04	3.47	43 ± 6
		3.5	59.9 ± 0.7	8.0	18.5 ± 1.0	7.0	2.07 ± 0.03	3.5	48 ± 2
		4.0	50.5 ± 0.4	9.0	15.9 ± 1.0	8.0	2.05 ± 0.03	3.8	42 ± 2
		5.0	41.7 ± 0.6	10.0	15.6 ± 0.7	9.0	1.94 ± 0.03	4.0	42 ± 3
		6.0	34.8 ± 0.5	15.0	12.5 ± 0.5	10.0	1.93 ± 0.02	5.0	32 ± 2
		7.0	31.5 ± 0.5	20.0	10.3 ± 0.2	15.0	1.74 ± 0.02	6.0	29 ± 2
		8.0	26.8 ± 0.4	25.0	9.0 ± 0.2	20.0	1.62 ± 0.01	7.0	26 ± 3
		9.0	24.3 ± 0.5	30.0	7.8 ± 0.2	25.0	1.53 ± 0.01	8.0	31 ± 4
		10.0	22.1 ± 0.2	35.0	7.1 ± 0.2	30.0	1.44 ± 0.01	9.0	24 ± 4
		11.0	20.0 ± 0.3			35.0	1.38 ± 0.01	10.0	18 ± 2
		12.0	18.8 ± 0.3					12.0	16.7± 1.4
		13.0	17.1 ± 0.2					15.0	12.2 ± 0.7
		14.0	16.3 ± 0.2					20.0	9.4 ± 0.7
		15.0	15.1 ± 0.2					25.0	7.1 ± 0.5
		17.0	13.5 ± 0.1					30.0	5.3 ± 0.3
		20.0	12.0 ± 0.1					35.0	5.0 ± 0.2
		25.0	9.7 ± 0.1						
		30.0	8.1 ± 0.1						
		35.0	7.1 ± 0.1						

Table S1 (follows).

NS2 dry		NS2+H_2O fast		NS2+H_2O slow		NS3 dry	
ω_L (MHz)	R_1 (s^{-1})	ω_L (MHz)	R_1 (s^{-1})	ω_L (MHz)	R_1 (s^{-1})	ω_L (MHz)	R_1 (s^{-1})
0.3	387 ± 11	0.1	38 ± 2	0.1	1.66 ± 0.05	0.7	218 ± 16
0.7	240 ± 4	0.3	38 ± 2	0.3	1.64 ± 0.03	1.0	181 ± 33
1.0	167 ± 3	0.7	32 ± 2	0.7	1.59 ± 0.03	1.3	134 ± 4
1.3	151 ± 3	1.0	38 ± 3	1.0	1.56 ± 0.02	1.5	115 ± 4
1.5	126 ± 3	1.3	38 ± 3	1.3	1.54 ± 0.02	1.7	100 ± 3
1.7	121 ± 2	1.5	38 ± 3	1.5	1.52 ± 0.02	2.0	81 ± 2
1.85	112 ± 2	1.7	36 ± 3	1.7	1.47 ± 0.02	2.1	85 ± 2
2.0	104 ± 2	1.85	34 ± 2	1.85	1.47 ± 0.02	2.2	78 ± 2
2.1	102 ± 2	2.0	34 ± 2	2	1.49 ± 0.02	2.3	77 ± 2
2.2	99 ± 2	2.1	37 ± 3	2.1	1.49 ± 0.02	2.35	79 ± 2
2.3	103 ± 2	2.3	36 ± 3	2.3	1.47 ± 0.02	2.4	74 ± 2
2.35	107 ± 2	2.35	38 ± 3	2.35	1.45 ± 0.02	2.45	85 ± 2
2.45	118 ± 2	2.4	37 ± 3	2.4	1.45 ± 0.02	2.5	75 ± 2
2.5	114 ± 2	2.45	37 ± 3	2.45	1.45 ± 0.02	2.55	76 ± 2
2.55	118 ± 2	2.5	37 ± 3	2.5	1.45 ± 0.02	2.6	80 ± 3
2.6	116 ± 2	2.55	37 ± 3	2.55	1.45 ± 0.02	2.7	74 ± 2
2.7	110 ± 2	2.6	37 ± 3	2.6	1.45 ± 0.02	2.8	75 ± 2
2.8	97.3 ± 1.3	2.7	36 ± 3	2.7	1.43 ± 0.02	2.9	69 ± 2
2.9	99 ± 2	2.8	34 ± 2	2.8	1.43 ± 0.02	3.0	67 ± 2
2.95	96 ± 2	2.9	36 ± 3	2.9	1.43 ± 0.02	3.05	61 ± 2
3.0	106 ± 2	2.95	36 ± 3	2.95	1.43 ± 0.02	3.1	69 ± 3
3.1	119 ± 2	3.0	37 ± 3	3	1.45 ± 0.02	3.15	70 ± 2
3.15	125 ± 3	3.1	37 ± 3	3.1	1.43 ± 0.02	3.2	65 ± 2
3.2	114 ± 2	3.15	36 ± 3	3.15	1.43 ± 0.02	3.3	65 ± 2
3.3	98 ± 2	3.2	34 ± 2	3.2	1.43 ± 0.02	3.4	60 ± 2
3.4	79 ± 2	3.3	33 ± 2	3.3	1.41 ± 0.02	3.5	52 ± 2
3.5	67.6 ± 1.3	3.4	31 ± 2	3.4	1.39 ± 0.02	4.0	45.0 ± 1.4
3.7	62.0 ± 1.0	3.5	30 ± 2	3.5	1.33 ± 0.02	5.0	35.4 ± 1.1
4.0	57.8 ± 1.1	3.7	30 ± 2	3.7	1.37 ± 0.02	6.0	25.8 ± 1.0
5.0	45.9 ± 1.0	4.0	28 ± 2	4.0	1.35 ± 0.06	7.0	26.1 ± 0.8
6.0	37.0 ± 0.8	5.0	25.0 ± 1.3	5.0	1.32 ± 0.02	8.0	19.5 ± 0.7
7.0	34.5 ± 0.7	6.0	23.3 ± 1.1	6.0	1.27 ± 0.02	9.0	18.2 ± 0.7
8.0	28.4 ± 0.8	7.0	22.7 ± 1.0	7.0	1.23 ± 0.02	10.0	13.8 ± 0.6
10.0	22.7 ± 0.7	8.0	20.8 ± 0.9	8.0	1.22 ± 0.01	11.0	12.7 ± 0.7
11.0	23.4 ± 1.0	10.0	18.2 ± 0.7	10.0	1.18 ± 0.01	12.0	10.6 ± 0.6
12.0	18.9 ± 0.8	11.0	17.2 ± 0.6	11.0	1.16 ± 0.01	13.0	10.4 ± 0.3
13.0	17.4 ± 0.4	12.0	15.9 ± 0.5	12.0	1.14 ± 0.01	14.0	9.7 ± 0.4
14.0	15.3 ± 0.2	13.0	15.6 ± 0.5	13.0	1.13 ± 0.01	15.0	9.2 ± 0.3
15.0	14.2 ± 0.2	14.0	14.9 ± 0.4	14.0	1.11 ± 0.01	17.0	8.4 ± 0.3
17.0	13.3 ± 0.2	15.0	13.9 ± 0.4	15.0	1.09 ± 0.01	20.0	7.3 ± 0.2
20.0	11.2 ± 0.1	17.0	13.2 ± 0.5	17.0	1.08 ± 0.01	25.0	5.81 ± 0.12
25.0	9.6 ± 0.1	20.0	12.2 ± 0.4	20.0	1.04 ± 0.01	30.0	4.98 ± 0.10
30.0	7.94 ± 0.07	25.0	10.8 ± 0.7	25.0	1.01 ± 0.03	35.0	4.26 ± 0.06
35.0	6.90 ± 0.05	30.0	10.3 ± 0.9	30.0	0.97 ± 0.03		
		35.0	7.8 ± 0.4	35.0	0.93 ± 0.03		

***iii*) Table S2. Halle's regression parameters R_1 vs ω_L curves.**

$$R_1 = \sum_i \frac{c_i \tau_i}{1 + (\omega_L \tau_i)^2} + \sum_{j(dips)} \left[\frac{2}{\pi} \cdot \frac{c_j s_j}{4(\omega_L - \omega_j)^2 + s_j^2} \right]$$

sample	c_i	τ_i (μs)	c_j	s_j (MHz)	ω_j (MHz)
βCD	209 ± 12	0.021 ± 0.003	-	-	-
	101 ± 10	0.31 ± 0.05	-	-	-
	94 ± 13	2.2 ± 0.6	-	-	-
NS1 dry	414 ± 8	0.027 ± 0.002	22 ± 2	0.33 ± 0.03	3.16 ± 0.01
	252 ± 13	0.20 ± 0.02	27 ± 4	0.57 ± 0.07	2.57 ± 0.02
	192 ± 10	1.60 ± 0.11			
NS1+H_2O	466 ± 9	0.0240 ± 0.0014	3.7 ± 1.4	0.5 ± 0.2	3.03 ± 0.05
"fast"	125 ± 7	0.233 ± 0.014			
NS1+H_2O	0.53 ± 0.06	2.9 ± 0.3			
"slow"	5.9 ± 0.5	0.202 ± 0.015			
	114 ± 7	0.0149 ± 0.0012			
NS1+D_2O	375 ± 12	0.060 ± 0.004	57 ± 28	1.6 ± 0.4	2.5 ± 0.2
"fast"	253 ± 21	1.2 ± 0.2			
NS2 dry	405 ± 12	0.021 ± 0.003	24 ± 2	0.30 ± 0.03	3.15 ± 0.01
	305 ± 14	0.19 ± 0.02	27 ± 4	0.48 ± 0.07	2.56 ± 0.02
	246 ± 14	1.48 ± 0.13			
NS2+H_2O	526 ± 16	0.0250 ± 0.0016	2.3 ± 0.8	0.27 ± 0.12	3.07 ± 0.03
"fast"	132 ± 8	0.188 ± 0.011	3 ± 2	0.5 ± 0.3	2.49 ± 0.07
NS2+H_2O	0.12 ± 0.06	1.2 ± 0.5			
"slow"	2.2 ± 0.2	0.19 ± 0.02			
	79 ± 7	0.0139 ± 0.0014			
NS3 dry	257 ± 26	0.018 ± 0.005	11 ± 6	0.6 ± 0.3	2.68 ± 0.06
	233 ± 33	0.22 ± 0.05	5 ± 3	0.3 ± 0.2	3.26 ± 0.04
	244 ± 39	1.5 ± 0.6			

iv) Normalized Inverse-Laplace (UPEN) T_1 distribution curves.

Figure S1. T_1 **distribution curves at 35 MHz.**

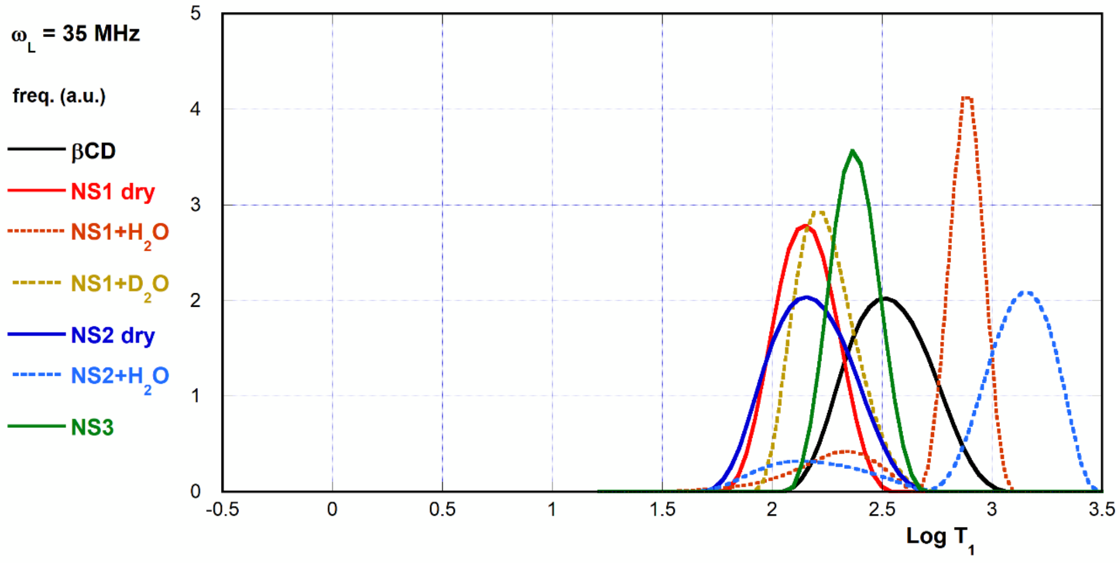

Figure S2. T_1 **distribution curves at 10 MHz.**

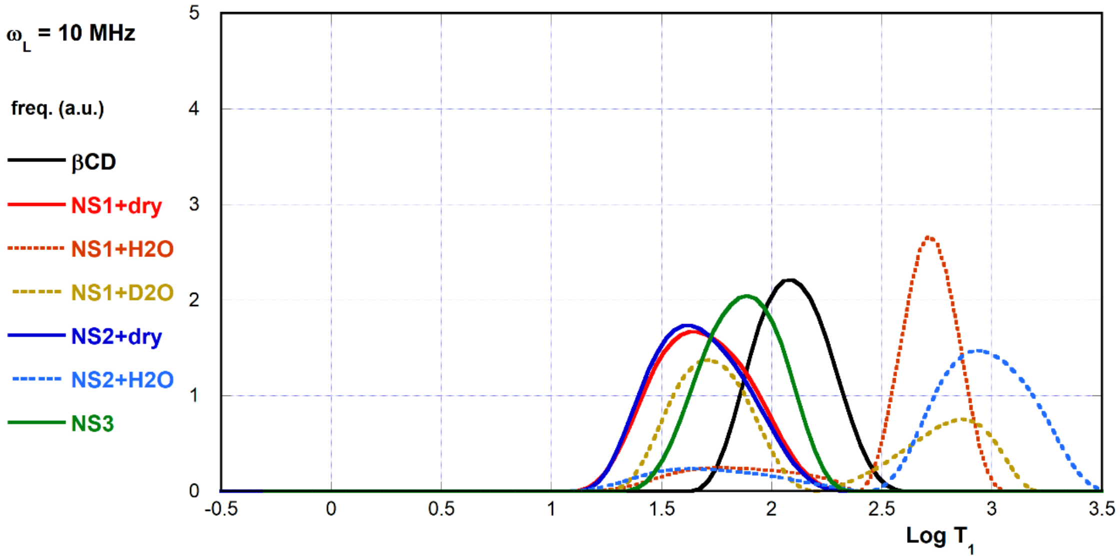

Figure S3. $\boldsymbol{T_1}$ **distribution curves at 3 MHz.**

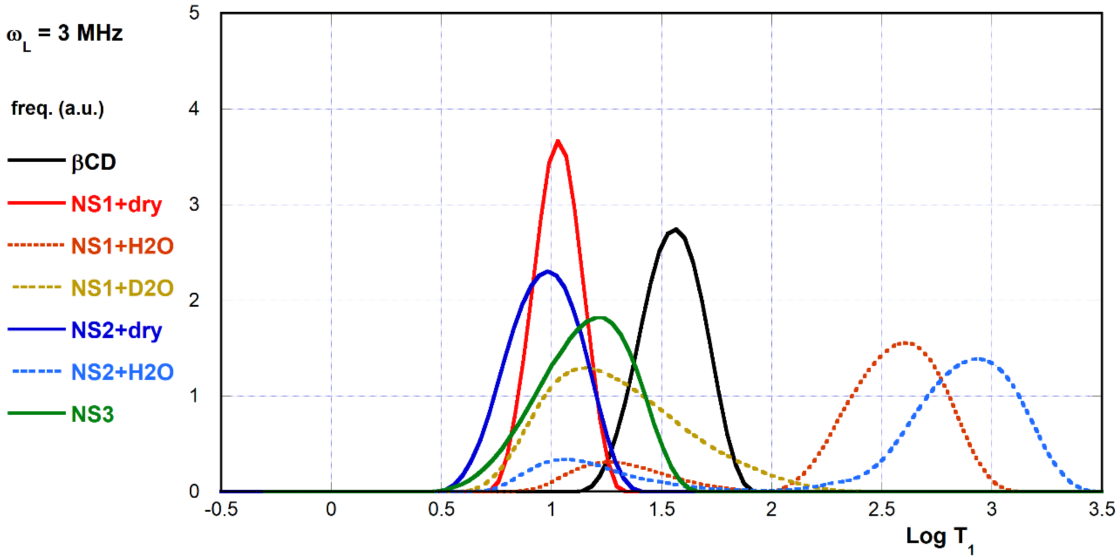

Figure S4. $\boldsymbol{T_1}$ **distribution curves at 1 MHz.**

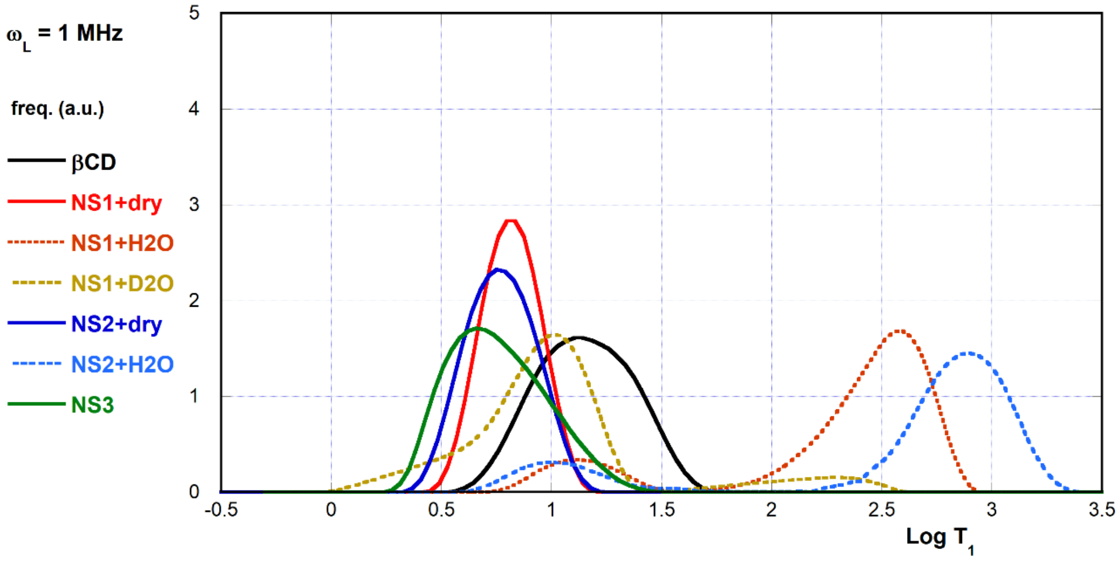

Figure S5. T_1 distribution curves at 0.3 MHz.

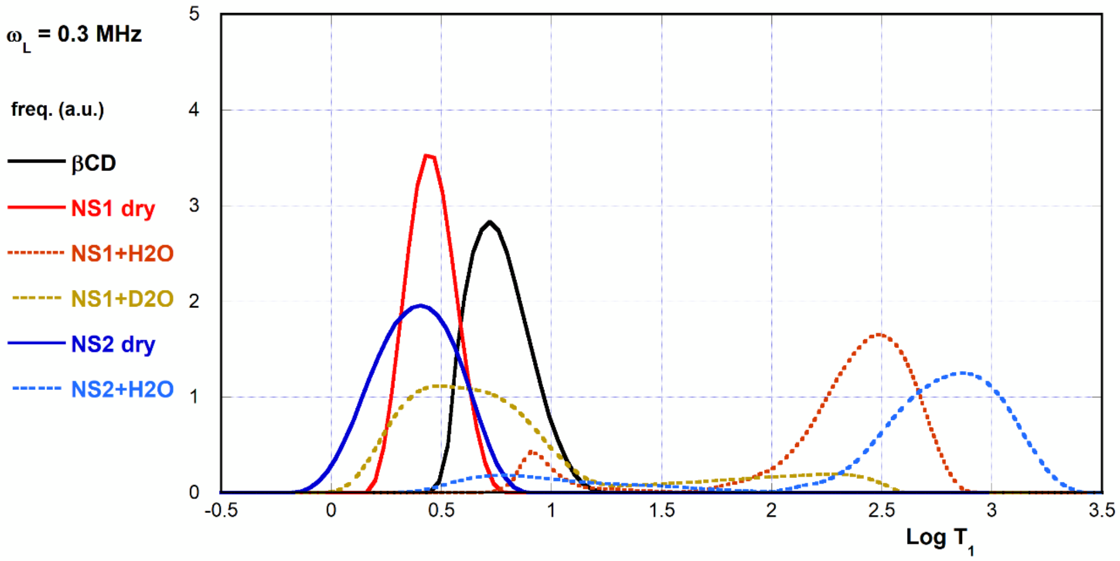

***v*) Table S3. Log-normal regression parameters for normalized Inverse-Laplace (UPEN) T_1 distribution curves.**

$$P(T) = \sum_i \left\{ \frac{1}{\sqrt{2\pi}} \cdot \left(\frac{p_i}{\sigma_i}\right) \cdot exp\left[-\frac{1}{2}\left(\frac{lnT - lnT_i}{\sigma_i}\right)^2\right] \cdot erf\left(a_i \cdot \frac{lnT - lnT_i}{\sigma_i}\right) \right\}$$

sample	ω_L (MHz)	p_i	σ_i	$\ln T_i$	a_i
βCD	35	1	0.239 ± 0.009	2.359 ± 0.012	0.74 ± 0.11
	10	1	0.209 ± 0.007	1.985 ± 0.011	0.62 ± 0.09
	3	1	0.177 ± 0.004	1.650 ± 0.006	-0.72 ± 0.06
	1	1	0.25 ± 0.04	1.09 ± 0.14	0.24 ± 0.56
	0.3	1	0.225 ± 0.003	0.601 ± 0.002	1.96 ± 0.09
NS1 dry	35	1	0.141 ± 0.001	2.151 ± 0.001	0
	10	1	0.296 ± 0.010	1.511 ± 0.013	0.81 ± 0.10
	3	1	0.113 ± 0.006	1.06 ± 0.02	-0.27 ± 0.17
	1	1	0.139 ± 0.001	0.816 ± 0.001	0
	0.3	1	0.112 ± 0.001	0.450 ± 0.001	0
NS1+H_2O	35	0.804 ± 0.005	0.086 ± 0.003	2.924 ± 0.005	-0.51 ± 0.08
		0.196 ± 0.003	0.315 ± 0.015	2.501 ± 0.008	-2.6 ± 0.4
	10	0.818 ± 0.012	0.121 ± 0.001	2.721 ± 0.001	0
		0.143 ± 0.011	0.27 ± 0.02	1.817 ± 0.017	0
	3	0.841 ± 0.009	0.276 ± 0.008	2.743 ± 0.008	-0.90 ± 0.08
		0.159 ± 0.010	0.29 ± 0.03	1.11 ± 0.03	1.3 ± 0.4
	1	0.849 ± 0.013	0.343 ± 0.001	2.745 ± 0.001	-2.48 ± 0.04
		0.151 ± 0.013	0.19 ± 0.02	1.04 ± 0.04	0. 5 ± 0.3
	0.3	0.912 ± 0.014	0.322 ± 0.004	2.659 ± 0.003	-1.8 ± 0.5
		0.088 ± 0.011	0.315 ± 0.010	0.850 ± 0.006	2.16 ± 0.07
NS1+D_2O	35	1	0.207 ± 0.002	2.098 ± 0.001	1.61 ± 0.05
	10	0.372 ± 0.007	0.318 ± 0.001	3.018 ± 0.005	-2.06 ± 0.18
		0.628 ± 0.008	0.178 ± 0.002	1.717 ± 0.002	0
	3	1	0.521 ± 0.003	0.917 ± 0.002	2.41 ± 0.05
	1	0.087 ± 0.011	0.45 ± 0.06	2.47 ± 0.03	-3.5 ± 1.8
		0.295 ± 0.015	0.152 ± 0.005	1.125 ± 0.006	0
		0.618 ± 0.014	0.235 ± 0.008	0.816 ± 0.016	0
	0.3	0.208 ± 0.003	0.90 ± 0.05	2.470 ± 0.005	-7.0 ± 0.7
		0.270 ± 0.014	0.187 ± 0.016	0.795 ± 0.018	0
		0.532 ± 0.013	0.32 ± 0.07	0.256 ± 0.005	1.62 ± 0.44
NS2 dry	35	1	0.238 ± 0.010	2.039 ± 0.013	0.74 ± 0.11
	10	1	0.318 ± 0.007	1.460 ± 0.006	1.16 ± 0.08
	3	1	0.205 ± 0.007	1.081 ± 0.010	-0.66 ± 0.08
	1	1	0.169 ± 0.001	0.768 ± 0.001	0
	0.3	1	0.200 ± 0.002	0.391 ± 0.002	0
NS2+H_2O	35	0.812 ± 0.008	0.206 ± 0.003	3.267 ± 0.005	-1.519 ± 0.02
		0.046 ± 0.014	0.11 ± 0.03	2.41 ± 0.05	0
		0.142 ± 0.012	0.18 ± 0.03	2.10 ± 0.04	0
	10	0.829 ± 0.009	0.273 ± 0.012	2.816 ± 0.016	0.76 ± 0.12
		0.052 ± 0.013	0.16 ± 0.06	2.00 ± 0.09	0
		0.127 ± 0.014	0.21 ± 0.05	1.60 ± 0.07	0
	3	0.818 ± 0.012	0.33 ± 0.06	3.110 ± 0.005	-1.21 ± 0.07
		0.192 ± 0.009	0.37 ± 0.02	0.884 ± 0.012	2.32 ± 0.45
	1	0.832 ± 0.014	0.294 ± 0.005	3.059 ± 0.004	-1.19 ± 0.06
		0.168 ± 0.012	0.30 ± 0.02	0.839 ± 0.014	1.47 ± 0.28
	0.3	0.859 ± 0.009	0.373 ± 0.008	3.055 ± 0.007	-1.27 ± 0.08
		0.141 ± 0.007	0.53 ± 0.08	0.55 ± 0.03	3.0 ± 1.2
NS3	35	1	0.125 ± 0.004	2.317 ± 0.007	0.48 ± 0.08
	10	1	0.233 ± 0.008	1.997 ± 0.011	-0.70 ± 0.09
	3	1	0.331 ± 0.004	1.384 ± 0.003	-1.57 ± 0.06
	1	1	0.378 ± 0.005	0.484 ± 0.003	2.10 ± 0.10

***vi*) Table S4. f_s vs ω_L data for NS1 and NS2.**

NS1		NS2	
ω_L (MHz)	f_s	ω_L (MHz)	f_s
0.1	0.097 ± 0.012	0.1	0.035 ± 0.003
0.3	0.086 ± 0.011	0.3	0.035 ± 0.005
0.7	0.075 ± 0.006	0.7	0.039 ± 0.003
1.0	0.068 ± 0.006	1.0	0.032 ± 0.003
2.0	0.069 ± 0.005	1.3	0.032 ± 0.002
2.1	0.068 ± 0.005	1.5	0.031 ± 0.002
2.2	0.070 ± 0.005	1.7	0.032 ± 0.002
2.3	0.069 ± 0.005	1.85	0.033 ± 0.002
2.4	0.067 ± 0.005	2.0	0.034 ± 0.002
2.45	0.071 ± 0.004	2.1	0.032 ± 0.002
2.5	0.071 ± 0.005	2.3	0.032 ± 0.002
2.6	0.064 ± 0.005	2.35	0.029 ± 0.002
2.7	0.070 ± 0.005	2.4	0.030 ± 0.002
2.8	0.072 ± 0.005	2.45	0.030 ± 0.002
2.9	0.063 ± 0.005	2.5	0.030 ± 0.002
3.0	0.066 ± 0.005	2.55	0.030 ± 0.002
3.05	0.063 ± 0.005	2.6	0.030 ± 0.002
3.1	0.063 ± 0.005	2.7	0.031 ± 0.002
3.15	0.071 ± 0.005	2.8	0.032 ± 0.002
3.2	0.070 ± 0.005	2.9	0.031 ± 0.002
3.3	0.067 ± 0.005	2.95	0.031 ± 0.002
3.4	0.070 ± 0.005	3.0	0.030 ± 0.002
3.5	0.074 ± 0.004	3.1	0.030 ± 0.002
4.0	0.081 ± 0.004	3.15	0.031 ± 0.002
5.0	0.088 ± 0.006	3.2	0.032 ± 0.002
6.0	0.090 ± 0.006	3.3	0.033 ± 0.002
7.0	0.096 ± 0.005	3.4	0.034 ± 0.002
8.0	0.095 ± 0.005	3.5	0.033 ± 0.002
9.0	0.103 ± 0.007	3.7	0.035 ± 0.002
10.0	0.105 ± 0.005	4.0	0.037 ± 0.003
15.0	0.116 ± 0.004	5.0	0.040 ± 0.002
20.0	0.129 ± 0.003	6.0	0.041 ± 0.002
25.0	0.138 ± 0.004	7.0	0.040 ± 0.002
30.0	0.149 ± 0.004	8.0	0.043 ± 0.002
35.0	0.153 ± 0.003	9.0	0.045 ± 0.002
		10.0	0.047 ± 0.002
		11.0	0.049 ± 0.002
		12.0	0.052 ± 0.002
		13.0	0.052 ± 0.002
		14.0	0.054 ± 0.002
		15.0	0.056 ± 0.002
		17.0	0.058 ± 0.002
		20.0	0.060 ± 0.002
		25.0	0.065 ± 0.005
		30.0	0.064 ± 0.006
		35.0	0.080 ± 0.005
